# Modeling Pharmacokinetics and Pharmacodynamics of Therapeutic Antibodies: Progress, Challenges, and Future Directions

**DOI:** 10.3390/pharmaceutics13030422

**Published:** 2021-03-21

**Authors:** Yu Tang, Yanguang Cao

**Affiliations:** 1 Division of Pharmacotherapy and Experimental Therapeutics, UNC Eshelman School of Pharmacy, University of North Carolina at Chapel Hill, Chapel Hill, NC 27599, USA; zoeytang@email.unc.edu; 2 Lineberger Comprehensive Cancer Center, School of Medicine, University of North Carolina at Chapel Hill, Chapel Hill, NC 27599, USA

**Keywords:** therapeutic antibody, PK/PD modeling, recycling antibody, bispecific antibody, tissue distribution, target engagement, effector functions, resistance

## Abstract

With more than 90 approved drugs by 2020, therapeutic antibodies have played a central role in shifting the treatment landscape of many diseases, including autoimmune disorders and cancers. While showing many therapeutic advantages such as long half-life and highly selective actions, therapeutic antibodies still face many outstanding issues associated with their pharmacokinetics (PK) and pharmacodynamics (PD), including high variabilities, low tissue distributions, poorly-defined PK/PD characteristics for novel antibody formats, and high rates of treatment resistance. We have witnessed many successful cases applying PK/PD modeling to answer critical questions in therapeutic antibodies’ development and regulations. These models have yielded substantial insights into antibody PK/PD properties. This review summarized the progress, challenges, and future directions in modeling antibody PK/PD and highlighted the potential of applying mechanistic models addressing the development questions.

## 1. Introduction

About a century ago, Paul Ehrlich proposed the ‘magic bullet’ concept, a drug selectively targeting a particular pathogen without affecting normal host cells [1]. This scientific concept became practical with the advanced engineering technologies to equip antibodies with high specificity to their cognate targets [2,3]. By 2020, more than 90 antibody drugs had been approved by the U.S. Food and Drug Administration (FDA) to treat a series of major diseases, such as autoimmune diseases and cancers [4]. There is no doubt that therapeutic antibodies have achieved significant clinical success and play central roles in revolutionizing many diseases’ treatment landscapes.

Immunoglobulin G (IgG) is the primary molecular format for the currently marketed therapeutic antibodies. IgG has high polarity and large molecular sizes (~150 kDa, approximately 14 nm) [5]. Those values are remarkably greater than small-molecule drugs that generally less than 0.9 kDa with sizes below 1 nm [6]. A full IgG consists of two antigen-binding fragments (Fabs), which recognize the cognate targets with high specificity, and one fragment crystallizable region (Fc), which binds to a range of cell-associated receptors such as neonatal Fc receptor (FcRn) and Fc gamma receptors (FcγR). The Fc-FcRn interaction plays a critical role in circumventing antibody catabolism and increasing antibody retention in the system, accounting for the antibody’s long half-life. IgG antibodies can engage the host immune system via interacting with FcγRs expressed in various effector cells. Those molecular properties greatly influence the pharmacokinetic (PK) and pharmacodynamic (PD) properties of therapeutic antibodies, endowing antibodies many therapeutic advantages such as long half-life, high potency, and limited off-target toxicity [6,7,8].

**Figure 1:**
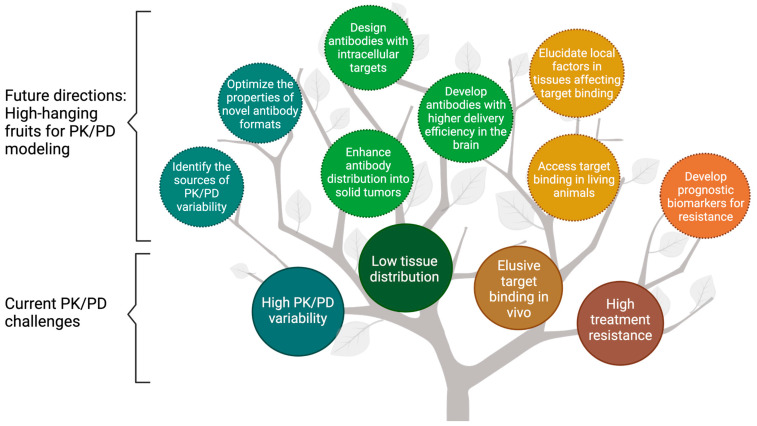
The fruit tree model presents the current challenges and future directions for modeling the pharmacokinetics/pharmacodynamics (PK/PD) of therapeutic antibodies. PK/PD modeling has been widely used to overcome the challenges associated with antibodies, including high PK/PD variability, low tissue distribution, elusive target binding in vivo, and high treatment resistance (enclosed in solid lines). This review discussed the future directions for PK/PD modeling in antibody development, namely the “high-hanging fruits” for PK/PD modeling (enclosed in dash lines). PK = Pharmacokinetics; PD = pharmacodynamics.

PK/PD analyses are integral to antibody development [7,8]. Antibodies PK studies are primarily focused on systemic persistence and antibody distribution in target tissues. Antibody PK influences the magnitude and duration of antibody PD. Antibodies elicit pharmacological actions through different modes of action (MoAs), including neutralizing pathogenic antigens, suppressing signaling pathways, or triggering effector functions [9]. Mechanism-based PK/PD modeling is a powerful tool to characterize the onset, magnitude, and duration of antibody treatment effects. Mechanistic PK/PD models are often valuable to elucidate the complex PK/PD relationships and shed light on factors determining intricate dose-response relationships. Numerous cases have demonstrated the successful application of PK/PD modeling to improve the efficiency and quality of antibody discovery, preclinical development, translational research, and decision-making in clinical development [10]. However, outstanding challenges remained in antibody PK/PD characterizations, including the high inter-subject variability, limited distribution into target tissues, unclear PK/PD properties of emerging antibody formats, flat dose-response relationships, and high resistance to antibody treatments (Figure 1). This article provides an overview of the progress, challenges, and future directions in characterizing antibody PK/PD properties and highlights the importance of applying mechanism-based PK/PD models in antibody discovery and development.

## 2. Modeling Pharmacokinetics of Therapeutic Antibodies

IgG antibodies’ systemic disposition is tightly associated with FcRn. FcRn is expressed in various cell types, such as vascular endothelium and hematopoietic cells [11,12,13]. Like endogenous proteins in the circulation, antibodies enter cells primarily via non-specific pinocytosis (e.g., fluid-phase endocytosis). Intracellular catabolism is the major elimination route for therapeutic antibodies [14]. While the lysosomal pathway catabolizes most proteins, a large proportion of IgG antibodies can be salvaged by FcRn. In early endocytic vesicles (pH 6–6.5), FcRn tightly binds to the antibodies’ Fc regions, protecting them from entering lysosomes. Bound antibodies are recycled back to the plasma membranes, where Fc-FcRn binding affinity decreases at a neutral pH (7.0–7.5). Antibodies are then disassociated from FcRn and released into the circulation. The FcRn-mediated antibody recycling protects approximately 90% of antibodies from catabolism and extends antibodies’ half-lives up to 20 days in humans [15]. Moreover, FcRn can carry the internalized antibodies across cells and release them into the basolateral side (e.g., tissue interstitium) [16]. Local variation in endothelial FcRn trafficking may significantly affect IgG tissue distribution, considering that a substantial fraction of total IgG resides in the extravascular space (~50%). However, the significance of FcRn-mediated transcytosis for antibody tissue distribution on the system level has not been convincingly demonstrated yet [15,17]. The majority of antibody enters tissue interstitium via convection [16,18]. In tissues with relatively large intercellular clefts on capillaries, antibodies can rapidly distribute to the interstitial space [19].

Many antibodies show rapid systemic clearance owing to target-mediated endocytosis, a phenomenon called target-mediated drug disposition (TMDD). Antibodies can extensively bind to their targets, forming antibody-target complexes, which are subsequently internalized and catabolized. Unlike the non-specific clearance via pinocytosis, target-mediated elimination is often capacity-limited and shaped by multiple factors, including antibody dose, target binding affinity, target expression, target turnover, and intracellular catabolism [16]. Many marketed antibodies exhibit dose-dependent elimination because of TMDD [16]. Modeling TMDD kinetics could yield insights into antibody PK, antibody-target binding kinetics, and antibody PD.

Although compartmental models are commonly applied in antibody PK analysis, they cannot provide many mechanistic insights into antibody PK. In contrast, physiologically based pharmacokinetic (PBPK) models offer an approach to characterize antibody PK in physiological and anatomical contexts. PBPK models have been widely applied to recapitulate many antibody PK behaviors, including tissue uptake and elimination [20], antibody-target binding in tissues [18], and FcRn-mediated antibody recycling [15]. Cao and Jusko reduced the full PBPK models into the minimal-PBPK (mPBPK) model, in which tissue compartments are grouped as “leaky” or “tight” based on their vascular structure and permeability [21,22,23,24]. The key determinants of antibody PK remained in the mPBPK models, such as convection as the major distribution pathway and the distribution space is limited in the interstitial fluid (ISF). The mPBPK models have been applied to characterize antibody PK profiles in various disease scenarios [25,26,27,28,29,30,31].

Despite the successful cases in which the antibody PK profiles were well-captured by the developed PK models, many ambiguities remain in antibody PK. For example, sources of large inter-individual variabilities and determinants of low tissue distributions are still not fully clear [32]. Moreover, PK properties of novel antibody formats, such as bispecific antibodies (bsAbs), are significantly different from conventional antibodies [8,33,34]. Understanding those complexities in PK is essential in developing and evaluating novel antibody products and improving antibody treatment efficacy and safety.

### 2.1. High Inter-Individual Variabilities

The unclear sources of high inter-individual PK variabilities make the treatment response greatly unpredictable. PK variabilities can arise from three aspects: patient characteristics and disease status, target properties, and anti-drug antibodies (ADA) [35,36]. The influences of demographic and anthropometric factors (e.g., weight and age) on antibody PK and the application of non-linear mixed-effects modeling to explore the sources of those inter-individual variabilities have been reviewed extensively [37]. This subsection will highlight the variabilities arising from target binding and immunogenicity.

The varying target expression can introduce PK variabilities through target-mediated elimination. High tumor burden (i.e., high target abundance) can lead to decreased half-life and low systemic exposure for many anti-tumor antibodies. A wide range of target baselines is often associated with highly variable antibody clearances and systemic exposures [38,39,40,41,42]. Target baseline has been commonly applied as a covariate to account for inter-individual variability in antibody clearance in PK/PD models [43,44,45]. Target baseline is often a time-dependent variable affected by disease status and progression, resulting in time-varying clearance and nonstable PK [35]. It is worth noting that target-mediated elimination, even sometimes without notable impact on systemic exposure, can potentially reduce antibody exposure at the target site, particularly when targets are in peripheral tissues where antibodies have limited local concentrations [46].

Another major source of variability for antibody clearance is ADA formation [47], a process affected by the interplay between factors related to the antibody itself (e.g., non-human sequence, glycosylation, impurities, aggregation), the patient (e.g., genetic factors, immune status), or the drug’s route and frequency of administration. ADAs can have multifold effects, including neutralizing the drug, altering antibody PK/PD profiles, reducing the treatment effect, or sometimes leading to severe adverse immune reactions [48,49,50]. Despite its high impact and prevalence, ADA’s molecular mechanisms are only partially understood [51]. The formation of ADA was primarily due to the non-human antibody portions recognized as “non-self” by the human immune system. Although most of the currently approved antibodies are humanized or fully human antibodies with reduced or absent non-human portions, which greatly reduced the ADA formation, the ADA incidences were still frequently observed across antibodies and populations [48,52,53,54,55]. In PK/PD models, ADA’s effect on antibody clearance is usually included as a dichotomous covariate [56,57] or a semi-quantitative covariate [58,59]. Notably, ADA’s concentration and avidity to therapeutic antibodies can shift over time, leading to time-varying effects on antibody clearance at the individual level. It remains challenging to make reliable predictions to ADA prevalence at the individual level, bringing uncertainly in predicting antibody PK [35].

Considering the high individual variabilities, monitoring the trough level of anti-TNFα antibodies, such as infliximab, has been applied in treating inflammatory bowel disease. Real-time anti-TNFα antibody measurements, coupled with Bayesian PK model prediction, can inform individual dosing regimens and improve clinical outcomes [60,61]. This approach is limited to a few anti-TNFα antibodies and not yet translated to other classes of antibodies.

### 2.2. Unique PK Properties of Novel Antibody Formats

**Figure 2:**
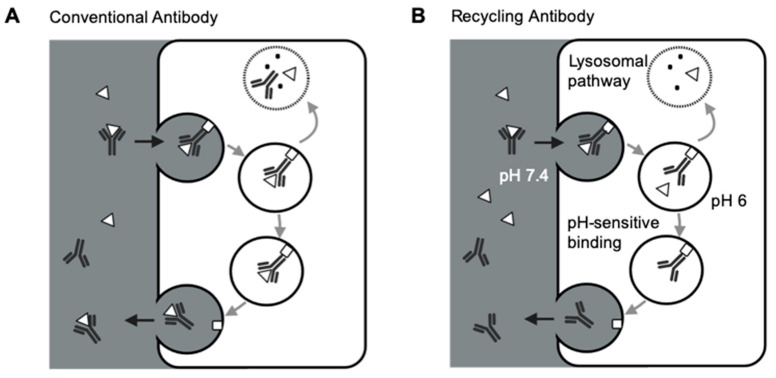
Schematic of FcRn-mediated recycling for conventional antibodies and recycling antibodies. (**A**) FcRn-mediated recycling for conventional antibodies. Conventional antibodies and the target-antibody complex are both salvaged and recycled back to the circulation by FcRn, making each antibody only has one chance of binding to the targeted antigen. (**B**) FcRn-mediated recycling for recycling antibodies. Recycling antibodies can disassociate the target antigens in the acidic endosomes due to the pH-sensitive antibody-target binding. FcRn recycles the antibodies, not the targets, making each antibody has more than one chance of binding to the targeted antigens. Δ: antigen; □: FcRn.

A number of engineering strategies have been applied to improve antibodies’ properties to achieve particular biological functions [62]. One of the most pursued goals for antibodies targeting pathogenic soluble targets is to improve target neutralization efficiency. In theory, each antibody only has one chance of binding to the targeted antigen [63]. FcRn salvage both free antibodies and antibody-target complexes, resulting in high antibody-mediated target accumulations (Figure 2A). Conventional antibodies often entail high doses and dosing frequencies to neutralize highly abundant pathogenic antigens. For example, eculizumab, a standard-of-care anti-C5 antibody in rare complement-dependent diseases, needs a dose of 900 mg every two weeks, which is about 10-time higher than other therapeutic antibodies, significantly increasing the financial burdens for the patients [64].

Several groups demonstrated the strategies of increasing FcRn binding to extend antibody half-lives and reduce antibody therapeutic doses [65,66,67]. However, such an approach is still limited by antibody-mediated antigen accumulation, restricting the targetable range of antigens for conventional antibodies. Igawa and colleagues have proposed the concept of recycling antibodies to overcome antibody-mediated antigen accumulations. The recycling antibody has a pH-dependent binding affinity to their cognate antigens, which decreases at pH 5.5–6.0 and remains mostly unchanged at pH 7.4 [63]. Therefore, the recycling antibodies can release antigens in the endosomes for lysosomal degradation, and the antibodies can be recycled back to the cell surface (Figure 2B). This approach can make antibodies bind to their pathogenic antigens multiple times [63]. Sweeping antibodies further improved the efficiency of removing pathogenic antigens by enhancing their affinities towards FcRn and increasing the antibody-antigen complex’s cellular uptake into endosomes [68,69]. Various clinical studies have demonstrated the safety and efficacy of such novel antibody formats [70,71,72]. For instance, ravulizumab, a sweeping antibody developed from eculizumab, had a terminal half-life 4-time longer than eculizumab. Ravulizumab achieved noninferiority for all efficacy end points when given to the patients every eight weeks compared with eculizumab given every two weeks [70,71].

Another engineering strategy to improve antibody functionality is to develop multi-specific antibody molecules. BsAbs have two binding domains for simultaneous binding to two different targeted antigens. BsAbs have a potential advantage over combination therapy, including synergistic efficacy and avoidance of treatment resistance [11,73]. Various bsAb formats were discovered and mainly categorized into IgG-like molecules and non-IgG-like molecules [73,74]. One popular concept of non-IgG-like bsAbs is bispecific T cell engager (BiTE), such as blinatumomab, the first BiTE approved by the FDA to treat acute lymphoblastic leukemia (ALL) [65,75]. Because of the absent Fc domain and smaller molecular sizes, blinatumomab has unique PK profiles compared to the conventional therapeutic antibodies. Blinatumomab does not undergo FcRn-mediated recycling, yielding a relatively shorter half-life (~ 2.1 h) than IgG-like bsAb emicizumab (~28 days) [75]. Blinatumomab redirects CD3 positive T-cells to CD19-expressing B cell malignancies, transiently connecting the two cells to induce T-cell-mediated killing of the bound B cell. Therefore, the efficacy of blinatumomab relies on the formation of immunological synapses between a cytotoxic T cell and a cancer cell, resulting in unique PK/PD and exposure-response relationships different from the conventional antibodies [76,77].

For those novel antibody formats with unique PK/PD profiles, mechanism-based PK/PD modeling can be valuable in their development, including providing rationales for antibody engineering and candidate optimization [78,79], offering insights into their MoAs [25,75,79,80,81,82,83], and facilitating their transition from preclinical space to the clinic [75,80,81]. For instance, to explore the inter-dependency of dual-target engagement, mechanistic target binding models were developed, which helped find the optimal binding affinity as a function of target isotypes, abundances, and cellular membrane properties. We provided a collective review about applying the modeling methods in characterizing PK/PD of novel antibody formats in the other sections.

## 3. Assessing Antibody Low Tissue Distribution

Restricted mainly in the vascular space, therapeutic antibodies have low distributions in tissues [18,84]. A clear exposure-response relationship is often evident for the antibodies with targets primarily in the peripheral blood. However, for antibodies with distal targets, their exposures at the site of actions are usually hard to characterize, obscuring exposure-response relationships. Evaluating antibody exposure at target sites is thus a critical task in antibody development and evaluation [85,86]. This section will introduce state-of-the-art tools for assessing antibody distribution in tissues and modeling antibody distribution determinants.

### 3.1. Tools for Evaluating Tissue Distribution

Numerous bioanalytical approaches have been applied to examine antibody distribution. Enzyme-linked immunosorbent assay (ELISA) [87,88], liquid chromatography-mass spectrometry (LC-MS) [17,89,90,91], and radioisotope quantification [17] are commonly applied to assess antibody tissue concentrations but unable to support longitudinal observations. There is a growing interest in using non-invasive approaches to monitor antibody tissue distribution continuously. Positron emission tomography (PET) has become a popular method to trace and monitor antibody tissue distribution in a spatiotemporal manner [92,93,94,95]. However, PET imaging is challenged by accumulated radiotoxicity and radionuclides’ short half-lives relative to the antibodies [96,97]. Other techniques such as fluorescence imaging are also frequently applied to access antibody distribution by detecting the signals emitted from the fluorophores conjugated to antibodies. Near-infrared (NIR) fluorescent probes such as IRDye800 have been applied widely in animal studies and clinical settings due to their enhanced tissue penetration and high target-to-background contrast [98,99,100]. One limitation in fluorescence imaging methods is the altered antibody disposition by fluorophore conjugation. The conjugation type and degree need to be optimized in order to improve the sensitivity while not altering antibody disposition [101].

It is worth noting that the total antibody tissue concentration is not the concentration at the site of action. The total antibody tissue concentration is merely a mixture of vascular, ISF, and intracellular antibody concentrations, which does not reveal the specific concentration at the target site concerning the spatial concentration gradient [102]. Antibody concentrations in the tissue ISF can be measured by preparing the interstitium fluid via ultrafiltration [103,104] or minimally invasive microdialysis [105,106,107]. Intravital microscope (IVM) imaging provides a high spatial and temporal resolution in assessing antibody distribution [108,109], making it a powerful tool for monitoring the spatial interactions between the antibodies and the target cells, characterizing antibody MoAs, and investigating underlying mechanisms of antibody treatment resistance.

### 3.2. Modeling Antibody Tissue Distribution

Extravasation of antibodies is primarily dominated by convective transport through paracellular pores and restricted by low net fluid rate and paracellular pores within the vascular endothelium [18,84]. A two-pore formalism theory was developed to characterize the convection of antibodies across the endothelium [110]. Covell et al. developed the first PBPK model for describing IgG antibody distribution into multiple tissues [20]. Baxter et al. included the two-pore formalism into the PBPK model to describe antibody convective transportation into tissues [19]. Many groups have spent tremendous effort in the past decade to incorporate different mechanistic factors in the PBPK model to predict antibody tissue distribution in a variety of scenarios [15,111,112,113,114]. The antibody biodistribution coefficient has been calculated by pooling together multiple sources of data in sought of a general agreement about antibody distribution extents across species and doses [112].

Although full PBPK models have been successfully applied to predict antibody PK and tissue distribution, our drug development community’s broad adoption of full PBPK models is still limited due to its complex structure and parameterization. In contrast, the mPBPK model provides a simple alternative to model antibody PK in a physiological context [21,23,24,103]. PBPK models are usually applied for more mechanistic exploration and species translation, while the mPBPK models could provide a simpler alternative at the systemic level but potential elaborations of tissue-of-interest. The mPBPK model has been adopted to assess the target binding dynamics in the target tissues and the effect of endosome trafficking on FcRn-mediated antibody recycling [23,25,26,27,28,30,115]. For example, Zheng et al. investigated the distribution of an anti-TNF antibody candidate CNTO5048 and its TNF-suppression effect by developing an mPBPK model with an extended compartment representing mice colon [26].

### 3.3. Emerging Antibodies with Intracellular Targets

Over half of approved drugs have intracellular targets [33,116,117,118]. However, those intracellular targets are generally not targetable for antibodies because antibodies cannot cross the cellular membranes [33]. Many approaches are explored to increase the antibody penetration into cells, such as delivery through virus-like particles or nanocarriers [117,119,120]. One of the most well-established methods for delivering antibodies into cells is intrabody-based gene therapy. In the intrabody strategy, the targeted cells are transfected or transduced by the gene encoding intrabodies to directly produced intrabodies within the cells. Intrabodies have been developed to treat various diseases, including cancer, neurodegenerative-disease, toxins, and viral infections. Deshane et al. endeavored this strategy to target intracellular erbB-2 protein. Unfortunately, although treatment effects were observed in vitro and in vivo studies [121,122], this intrabody therapy did not achieve significant clinical efficacy [123]. The lack of treatment efficacy could be partially associated with the unclear PK/PD profiles [124,125,126]. For instance, the pre-existing immune response against adenovirus could influence the PK/PD profiles of the gene therapies with adenoviral vectors and diminish transgene expression efficiency [127]. A better understanding of the apparent PK-PD discrepancy is sorely needed to elucidate the intracellular exposure and the final efficacy of gene transcription.

Another strategy to deliver therapeutic antibodies into cellular compartments is to fuse antibodies with autoantibody fragments for their intrinsic ability to enter cells. Autoantibodies are naturally occurring and target host-self intracellular or intranuclear antigens during autoimmune diseases [128], possessing an intrinsic ability to penetrate living cells and interact with DNA, histones, and ribosomal. One of the proof-of-concept antibody products is cytotransmab [129,130]. Koi et al. demonstrated the potential of cytotransmab for entering into living cells through a clathrin-mediated endocytic pathway, directly targeting cytosolic proteins [129]. The PK/PD properties of these novel therapeutic agents remain unclear. Mechanistic PK/PD models focusing on intracellular transportation kinetics will be critical to informing the drug development and supporting better clinical translation.

### 3.4. Antibody Distribution in Solid Tumors

**Figure 3:**
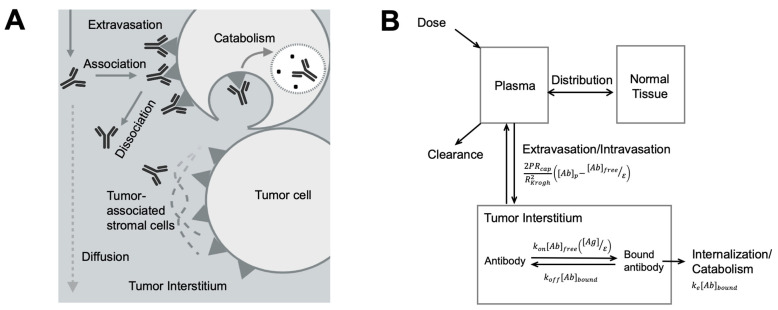
Antibody spatial distribution in tumors. (**A**) Target interaction and tumor microenvironment influence antibody spatial distribution. After extravasation into the tumor interstitium, antibodies rapidly associate with its targets in perivascular tumor cells, forming a binding site barrier and limiting its diffusion to the deeper tumor. Antibodies bound to the targets can be endocytosed and degraded. Other components, such as tumor-associated stromal cells, also hinder antibody interaction with its targets and antibody diffusion. (**B**) A simplified antibody spatial distribution model in tumors. The plasma, normal tissues, and the tumor were included in a simplified Krogh Cylinder model. The arrows indicated antibody movements between compartments. The arrows indicated antibody movements between compartments. The antibody extravasation was a function of *P* (the permeability coefficient for antibody across the tumor capillary wall), *R*_cap_ (the capillary radius in the tumor), *R*_Krogh_ (the average radius of tissue surrounding each blood vessel), [*Ab*]_p_ (antibody concentration in the plasma), [*Ab*]_free_ (free antibody concentration in the tumor), and ε (void fraction). In the equation describing antibody binding, *k*_on_ is association rate constant, *k*_off_ is disassociation rate constant, [*Ag*] is the target concentration in the tumor, and [*Ab*]_bound_ is the concentration of bound antibodies. Bound antibody internalization is described by the degradation rate constant *k*_e_. (Adapted from Figure 1A [137], Elsevier, 2012).

Solid tumors are abnormal and heterogeneous tissue consisting of various cell types (Figure 3A). The constant interplays between malignant cells, immune cells, blood, and lymphatic vessels, and tumor-associated fibroblasts compose the highly dynamic and heterogeneous tumor microenvironment (TME) [131]. Larger pore sizes, varying diameters, and irregular branching patterns are often observed in tumor blood vessels, leading to the abnormal blood supply in solid tumors [132,133]. Necrotic regions develop due to the lack of functional blood vessels in solid tumors and the inefficient delivery of oxygen and nutrients. The complex vascular and extracellular contents in solid tumors significantly influence antibody spatial distribution, resulting in limited and variable target accessibility and suboptimal treatment effect [134]. Furthermore, due to the lack of lymphatic vessels, many blood macromolecules were leaked out of the vessel and stuck in the tumor, causing high interstitial hydrostatic pressure and further restricting antibody diffusion in the tumor bed. The effective diffusion coefficient of antibodies in solid tumors is as low as 1.3 × 10^−8^ cm^2^/s [135], denoting that antibodies need more than one day to diffuse 1 cm within the tumor bed [136].

Compared to the slow diffusion rate, the antibody-target association rate is relatively faster. Such a rapid and extended antibody-target binding process can become a barrier for antibodies diffusing into deeper tissues, creating a barrier known as the “binding site barrier” (Figure 3A) [137], which is the major reason for perivascular antibody distribution in solid tumors. Many other TME components can also influence spatial antibody distribution in solid tumors [138,139]. For instance, stroma cells usually grow around tumor cells, giving rise to a dense tumor matrix network, creating a physical barrier for antibody distribution within tumors [140]. The stress stroma can restrict antibody diffusion leading to the accumulation of antibodies at stroma-rich areas [141,142,143,144].

Agent-based modeling methods have been applied to account for antibody spatial-temporal distribution in tumors. In an agent-based model, individual discrete agents can represent antibodies or the diverse cell populations that interact with each other under defined rules. A tridimensional heterogeneous TME can be constructed by moving along a 3D lattice, providing real-time simulations of antibody diffusion in TME and the resultant cellular responses [145,146,147,148,149]. For example, Kather et al. designed a 3D agent-based model of human colorectal cancer, which includes TME components such as lymphocytes and fibrotic stroma, to evaluate treatment effects of immunotherapy [148]. Menezes et al. developed a hybrid agent-based model to capture antibody delivery in solid tumors for predicting tumor killing and growth kinetics [149].

Models involving ordinary or partial differential equations were developed to account for antibody concentration gradients within tumors [136,150,151,152,153]. A simplified spatial distribution model derived from the Krogh cylinder model is broadly applied to understand the dynamic interplays between antibody extravasation, diffusion, target affinity, and internalization. Antibody perivascular distribution and the influencing factors are well explained using the simplified model (Figure 3B) [137]. As promising as they are, these models could be challenging to gain full validation, limiting their application into making clinical predictions.

### 3.5. Antibody Distribution in the Brain

The brain is a notorious tissue for antibody targeting. The tight junctions between capillary endothelial cells create a physical barrier for antibodies to penetrate [154]. The brain’s antibody concentration is only ~0.1% of that in the peripheral blood [16]. The limited antibody distribution in the brain is the primary challenge in developing antibodies for neurodegenerative disorders, such as Alzheimer’s disease and Parkinson’s disease. This subsection will discuss the factors that significantly influence antibody brain penetration and the modeling and simulation approach in elucidating antibody brain disposition, and the current strategies to increase antibody brain disposition.

Antibodies enter the brain parenchyma primarily via blood–brain-barrier (BBB) or enter the cerebrospinal fluid (CSF) via blood-CSF-barrier (BCSFB). Antibodies can cross those barriers through receptor-mediated transcytosis (RMT) [155]. In the RMT, antibodies can bind to the transmembrane receptor on the apical plasma membrane and are subsequently endocytosed and trafficked to the basolateral plasma membrane, where the antibodies are released into the brain. Various receptors can participate in RMT, including transferrin receptors (TfR), insulin receptors (IR), and low-density lipoprotein receptors (LDLR). The role of FcRn in the RMT is still unclear for antibody brain penetration [111,156,157,158,159,160].

Tremendous effort has been invested in improving antibody delivery into the brain [161,162,163,164]. For instance, Kinoshita et al. introduced a technique to increase antibody brain delivery through transiently disrupting BBB by ultrasound, opening up tight cellular junctions, and facilitating the antibody penetration in the brain [164]. RMT-based antibody delivery has gained momentum as a viable method to treat central nervous system (CNS) disorders [78,165,166,167,168]. Most of our RMT-based antibody delivery experience was gained from anti-TfR antibodies, which significantly increased antibody brain uptakes compared to the conventional antibodies [78,169]. There is a tradeoff between antibody affinity to TfR and RMT efficiency. A very-high anti-TfR1 affinity would alter the TfR trafficking and make antibodies trapped in endosomes, reducing RMT efficiency. A bell-shaped relationship between TfR affinity and antibody brain exposure has been well documented [78,169]. Yu et al. developed a series of bsAbs to target TfR and beta-secretase 1 (BACE1) with different affinities to TfR [78]. The bsAb variant with a relatively lower affinity to TfR had the highest brain exposure than the other variants. These pieces of evidence suggest the potential of applying modeling methods in optimizing the brain-delivery efficiency of the RMT-based antibodies.

The complex and dynamic biofluid system can substantially influence antibody distribution kinetics in the brain. The fluid filtered by the cerebral blood vessels joins in the cerebral ventricles, beginning CSF circulation. A total of 150 mL CSF fluid are present in an adult human brain. CSF bulk flow is approximately 24 mL/h, continuously replacing CSF as it is absorbed [170,171]. It is generally believed that CSF flow plays a major role in antibody infiltrations into the CSF [172]. However, many studies suggested that antibody infiltration rate in the CSF could be significantly lower than the CSF bulk flow [173,174,175]. The glymphatic system, a collection of perivascular spaces promoting fluid exchange between CSF and brain ISF, plays a potential role in the convective transportation of antibodies from the CSF to the brain parenchyma, which could be affected by brain diseases [175,176,177,178,179,180]. The involvement of the glymphatic system in antibody brain distribution warrants further exploration, and engineering antibodies to target this system may represent a future research direction.

The technical and ethical challenges for directly sampling and measuring antibody brain concentrations make mathematical modeling a helpful tool for understanding antibody brain distribution [171,181]. Chang et al. developed a whole-body PBPK model to describe a non-specific antibody’s spatial distribution in multiple brain areas and the distribution kinetics associated with brain biofluid flow [175]. We have characterized the brain distribution kinetics of anti-α-synuclein antibody candidates using an mPBPK model with an extended CSF compartment [182]. Our study further showed that antibody penetration rate into the CSF is significantly lower than the CSF bulk flow.

## 4. Elucidating Antibody-Target Engagement

Antibody-target engagement is the first step to elicit the cascade of the pharmacological action. The magnitude of antibody-target engagement can serve as a critical biomarker for selecting therapeutic doses and predicting therapeutic effect. The tools for measuring antibody-target engagement, the models that describe antibody-target binding kinetics, and the applications of the modeling methods for antibody candidate optimization will be discussed in this section.

### 4.1. Measuring Antibody-Target Engagement

The measurements of antibody-target engagement can be at either microscopic or macroscopic levels. Flow cytometry (FCM), Immunohistochemistry (IHC), and immunofluorescence (IF) staining can provide a time-frozen snapshot of target engagement on either the circulating cells or tissue-derived cells [183,184]. At the macroscopy level, radiotracer replacement studies are often conducted to measure target engagement based on the competitive binding between small doses of radiolabeled antibodies and increasing amounts of cold antibodies. However, rapid endocytosis of radiotracers and the residualized isotopes can introduce bias to such measurements. Although PET/single-photon emission computed tomography (PET/SPECT) and fluorescence imaging methods monitor antibody distribution and tissue-specific target engagement in a continuous manner [185,186], they cannot differentiate the signal of free antibodies from the bound antibodies, precluding the accurate estimation of target engagement. A non-invasive imaging method to directly monitor target engagement with temporal and spatial resolutions is desirable.

**Figure 4:**
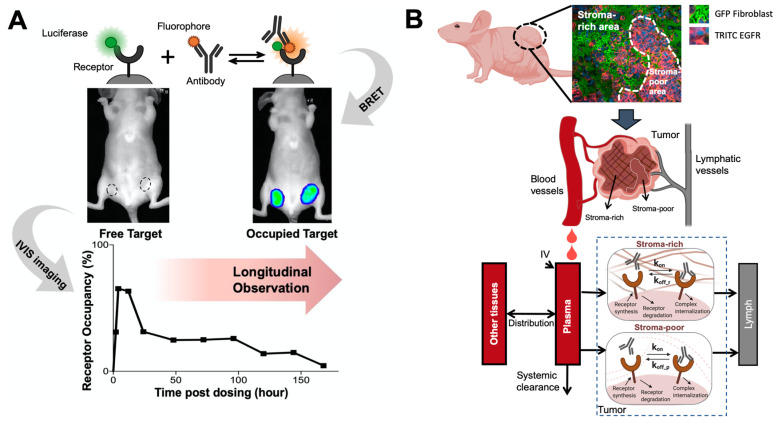
Elucidating antibody-target binding dynamics in the living system. (**A**) A bioluminescence resonance energy transfer (BRET) imaging method continuously and noninvasively monitor antibody-target interactions in the living tumors. A bright luciferase, NanoLuc, was fused to EGFR as the BRET energy donor. When cetuximab binds to EGFR in solid tumors, the distance between stimulated NanoLuc and DY605 (fluorophore) enables the resonance energy transfer from stimulated NanoLuc to DY605-cetuximab. DY605 emissions from living tumors directly visualized the interactions between cetuximab and EGFR (Adapted from abstract graph [193], Cell Press, 2019). (**B**) A spatially-resolved model characterizes antibody-target binding dynamics in living tumors. The tumor is divided into two compartment (the stroma-rich vs. stroma-poor tumor area), and the model assumes antibody binds to its target at different dynamics in two compartment, which well recapitulated the longitudinal imaging data (Adapted from Figure 2 [194], Nature Research, 2020).

Proximity-based imaging technologies, including the Förster resonance energy transfer (FRET) and bioluminescence resonance energy transfer (BRET), recently showed promise to provide a direct assessment of antibody-target engagement. Those technologies detect antibody-target interactions upon the energy transfer between the antibody and the receptor once both are in proximity. In a FRET pair, the energy donor is usually a fluorophore that can be excited by monochromatic light [187,188], while the donor is often a luciferase in a BRET pair [189]. The energy acceptor in either FRET or BRET pairs are fluorophores, which usually emit light at a different wavelength to avoid signal interference. When the donor-to-acceptor distance allows the resonance energy transfer, the acceptor will re-emit the light, directly indicating the interactions between the donor and the acceptor [190,191]. BRET has several advantages over FRET, such as the lack of photobleaching, making it applicable for long-term monitoring. We recently developed a BRET antibody-target pair to investigate antibody-target binding dynamics in living tumors (Figure 4A). In this study, a bright luciferase, NanoLuc [192], was fused to EGFR as the BRET energy donor. A fluorophore, DY605, was covalently conjugated to anti-EGFR antibody cetuximab. When cetuximab binds to EGFR in solid tumors, the distance between stimulated NanoLuc and DY605 enables the resonance energy transfer from stimulated NanoLuc to DY605-cetuximab. DY605 emissions from living tumors directly visualized the interactions between cetuximab and EGFR [193]. These proximity-dependent sensing approaches have become extremely attractive for quantifying antibody-target engagement, supporting continuous monitoring of antibody-target interactions.

### 4.2. Modeling Antibody-Target Binding Dynamics

Antibody-target engagement is not only a critical step in antibody dispositions but also influences pharmacological actions. For antibodies with a high target abundance, the extensive target binding and the rapid internalization could confer nonlinear PK behaviors to antibodies, known as TMDD [195]. Many antibodies targeting transmembrane antigens frequently exhibit TMDD behaviors [195]. TMDD models can provide a mechanistic bridge between antibody PK and the pharmacological responses, depicting the intricate interactions among antibodies, targets, and anti-target complexes. Since Mager and Jusko proposed the first TMDD model [196], many delicate TMDD models have been developed [195].

Incorporating the TMDD models into the mPBPK model provides a unique aspect for characterizing antibody-target interaction at the site of actions and investigating the local environment-specific binding properties [23]. Cao and Jusko developed the first mPBPK model extended with TMDD to assess target bindings properties in either the plasma or tissue ISF compartment [23]. This model offers the chance to elucidate antibody-target binding properties at different tissue contexts. For instance, we recapitulated the PK and target suppression profiles of two anti-α-synuclein antibody candidates in the peripheral blood and the CSF using an mPBPK model with an extended CSF compartment. We found that an antibody could have distinct binding dynamics at different anatomical sites due to local physiological environments’ influences.

Antibody-target binding parameters including *k*_on_, *k*_off_, and *K*_D_ are usually measured using in vitro binding assays such as surface plasmon resonance in static environments [67,197]. Amounting evidence has indicated that these in vitro methods lack in vivo correlation due to the static non-native in vitro binding conditions not revealing the physiological factors [198,199,200,201,202], such as pressure and shear force in the living tissues [203]. The modeling approaches can be a complementary and powerful tool for identifying the in vivo binding parameters and exploring the underlying mechanisms that affect antibody-receptor binding in the living system [204]. For instance, Li et al. investigated the impact of tissue-specific ISF turnover rates on the binding kinetics between antibodies and soluble targets [115]. In the tissues with low ISF turnover, antibodies with a relatively lower *k*_off_ can achieve a greater target suppression. Antibodies with a high *k*_on_ are favored in the tissues with high ISF turnover. Those findings explained why etanercept showed relatively higher treatment efficacy in rheumatoid arthritis than in Crohn’s disease, as the relatively high *k*_on_ allows etanercept to have higher efficacy in suppressing the TNF in joint synovium than in the colon.

Physical structures and restrictions in the target tissues can also alter the target binding dynamics, especially in solid tumors. Tumor stromal cells can cause spatial hindrance and mechanical stress in solid tumors, influencing the dynamics of antibody-antigen interactions. We developed a spatially-resolved computational model to characterize cetuximab-EGFR binding kinetics and compare it between the stroma-rich area and the stroma-poor area within solid tumors [194] (Figure 4B). Restricted diffusion of cetuximab in solid tumors makes cetuximab-EGFR binding to a slower-but-tighter degree in the living tumor compared to the in vitro systems. Compared to the tumor regions that lack stroma cells, cetuximab had a slower disassociation rate constant in the stroma-rich areas, which was further confirmed in the immunofluorescent staining showing that a high fraction of cetuximab stayed bound in the stroma-rich tumor regions. These studies demonstrated the essential role of modeling in elucidating antibody-target binding dynamics in physiological contexts.

### 4.3. Optimizing Target Binding Affinity

TMDD models have been widely applied in model-informed drug development of therapeutic antibodies. Antibody-target binding kinetics can be considered in the PK/PD models to predict the desirable antibody properties [205,206]. Antibodies with a high target affinity are desirable in the early stage of development for achieving a high and durable target coverage [207]. However, this is not always the case to select antibodies with the highest affinity [208]. The optimal antibody affinity should be made by considering multiple factors, including the target properties, antibody disposition at the site of action, and MoAs [209].

For antibodies acting through neutralizing soluble ligands or suppressing membranous signaling, increasing affinity may not always enhance treatment effects. Tiwari et al. demonstrated that the target baseline concentrations significantly affect the optimal *K*_D_ for antibodies neutralizing soluble targets [208]. In contrast, the optimal *K*_D_ for antibodies suppressing membranous signaling is contingent antibody-target complex internalization rate. Concerning almost all of the current antibody products having their reported *K*_D_ values falling within the optimal range, this study suggested that when developing an antibody candidate with the same target and MoA as a marketed antibody, optimizing *K*_D_ is less necessary to improve its treatment outcomes [208]. Many other studies achieved similar conclusions [10,210,211,212]. Agoram et al. demonstrated that a decrease in the *K*_D_ of omalizumab, an anti-IgE antibody, did not increase treatment efficacy [10]. Penney and Agoram later observed that K_D_ values of current antibody products only affect treatment effect to a limited extent due to the influences of many other PK and target-associated parameters [210]. Overall, those findings suggest that the target affinity should be assessed along with many other PK/PD parameters. Using PK/PD modeling to identify optimal K_D_ values can avoid the wasteful investments in multiple cycles of affinity maturation to generate high-affinity antibodies.

Antibody binding properties are also associated with antibody tissue penetration and retention [209]. An inverse relationship between antibody-target binding affinity and antibody spatial dispersal has been widely observed. For instance, single-chain Fv antibody molecules with high affinity may confer high endocytosis and shorter durations of antibody-target complex on the cell membrane, leading to greater degradation and limited tumor penetration [213]. Antibodies with moderate affinities could diffuse more widely than those with high affinities [213]. Similar findings were reported by Adams et al. [212]. PK/PD modeling can help investigate the desired binding properties to achieve optimal PK profiles. Gadkar et al. tested different variants of anti-TfR/BACE1 antibodies and found the variant with a higher affinity to TfR showed a higher systemic clearance and a lower brain uptake. The PK/PD profiles of the bsAb candidates with a range of affinities to TfR were further simulated to determine the optimal affinity and guide candidate selection [214].

**Figure 5:**
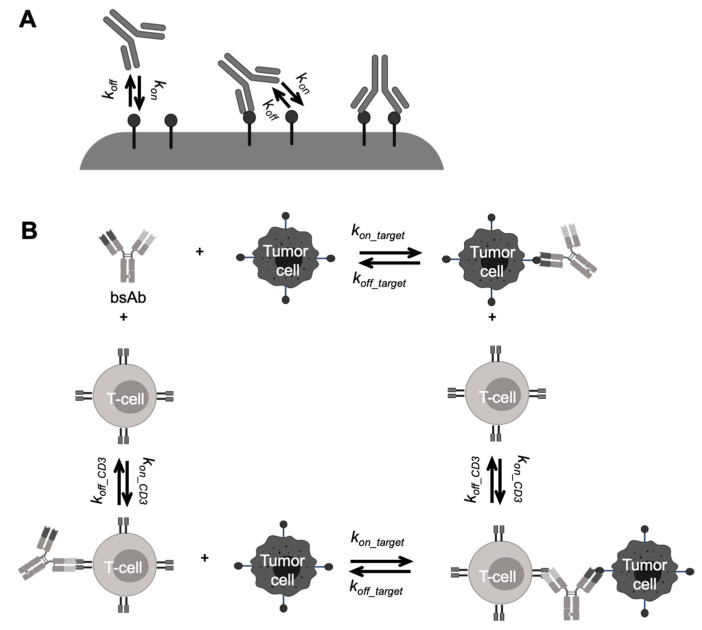
Schematic of antibody-target binding models for bispecific antibodies (bsAbs). (**A**) Schematic of multivalent antibody binding model. The two arms of a bsAb separately bind to targets with the monovalent binding parameters (*k*_on_ and *k*_off_). Upon the first binding, the second one while on the cell surface, subject to rate-limiting lateral diffusion within the lifetime of the first engaged antibody-antigen complex (Adapted from abstract graph [77], American Society for Biochemistry and Molecular Biology, 2016). (**B**) A mechanism-based model describing bispecific T-cell engager (BiTE) binding kinetics. The free BiTE binds to a T-cell or a tumor cell independently, forming BiTE-cell dimers. The dimers further bind to either T-cells or tumor cells and form trimmers, which drive the pharmacodynamic effects of BiTEs (Adapted from abstract graph [76], Taylor & Francis, 2018).

Modeling approaches can also help determine the binding properties associated with the optimal pharmacological effects of novel antibody formats. A certain threshold of target expression is required for the optimal avidity of bsAbs, which can be different for each antigen [215,216]. Simultaneous binding of both bsAb arms will only happen when the second receptor is in the first receptor’s vicinity, which greatly influences the avidity of bsAbs. Rather than assuming each arm of a bsAb binding to targets independently with monovalent binding kinetics parameters (Figure 5A), many modeling studies have provided insights into improving bsAb avidity by simulating the antibody crosslinking events with different individual arms’ affinities and target expression levels [77,217]. The influences of the spatial limitation on bsAb bivalent binding were also evaluated in mechanism-based models, either on the same cell surface [218,219,220] or on different cell surfaces [221,222]. Those studies provided a deeper understanding of the factors that regulate bsAb dual-targeting properties to aid bsAb engineering and selection efforts.

Another well-known example of using modeling to optimize binding properties is BiTE, whose treatment effects are more dependent on the synapse formation. A very high affinity to CD3 can result in monovalent binding, leading to premature activation of T cells and accumulations of antibodies in the CD3-rich tissues [223]. PK/PD modeling can help evaluate optimal target affinity in different local tissue environments for decision-making in the early stage of antibody development [76,79,208,224,225,226,227,228]. For example, Jiang et al. developed a trimer-based cell-killing model to describe blinatumomab PK/PD profiles in ALL patients [76]. In this model, the independent BiTE-CD3 and BiTE-CD19 binding events were described by two TMDD models, separately determined by CD3 or CD19 densities, and BiTE-CD3 or BiTE-CD19 binding affinities. The BiTE-T-cell and BiTE-tumor-cell dimmers had further binding events to form trimers eliciting the tumor lysis (Figure 5B) [76]. This model reasonably described multiple sets of in vitro cytotoxicity data, and adequately predicted the influence of several key factors on the in vitro cytotoxicity assays and clinical effective dose of blinatumomab.

**Figure 6:**
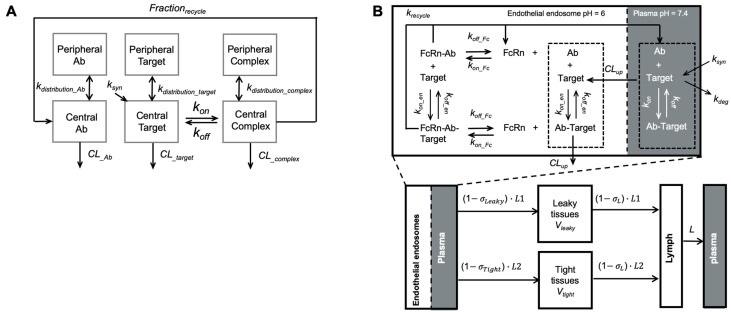
Modeling recycling antibodies. (**A**) A target-mediated drug disposition (TMDD) modified to include a fraction of recycling. The model included the distribution kinetics of recycling antibodies, pathogenic targets, and antibody complexes between the central and peripheral compartments. The antibody-target binding was only considered in the central compartment. F_rec_ described the FcRn-mediated recycling, the fraction of antibody recycled from endosome (Adapted modified from Figure 1 [81], Elsevier, 2016). (**B**) A minimal-PBPK model with a nested endosomal compartment describing the binding kinetics of recycling antibodies. The antibody-target binding was included in both plasma (shadowed) and endosome compartments. FcRn-mediated antibody recycling was considered in the endosome compartment. (Adapted from Figure 1 [25], Springer Science, 2018).

Model-based PK/PD models have also been applied to optimize the sweeping and recycling antibodies’ binding properties and antigen removal efficiencies (Figure 6). In addition to conventional compartmental TMDD models mechanism-based models were developed to provide mechanistic insights into the binding kinetics of recycling antibodies (Figure 6A). Our group investigated the effects of antibody-FcRn association rate, disassociation rate, and endosomal transit time on the antibody clearance in different species using an extended mPBPK model [82]. The study indicated that the increase in antibody-FcRn binding affinity is beneficial for extending recycling antibodies’ systemic persistence [82]. The impact of antibody-FcRn disassociation rate on antibody systemic persistence is restricted by endosomal transit [82,113]. Yuan et al. further demonstrated this point with a more mechanistic elaboration on the antibody-FcRn and antibody-target binding in an endothelial endosome compartment, providing a platform for evaluating the PK and disposition behaviors of Fc-engineered antibodies and recycling antibodies (Figure 6B) [25]. An extended model structure with a second endothelial endosome compartment was later developed for describing the membrane target-mediated antibody target-mediated of concizumab, a recycling antibody that targets tissue factor pathway inhibitor in both soluble and membranous forms [83]. This work highlighted the influences of target release in the endosomes on antibody clearance and systemic persistence [83]. In summary, those examples emphasized that PK/PD modeling the antibody-target binding can be invaluable in the design and development of therapeutic antibodies.

## 5. Modeling Pharmacodynamics of Therapeutic Antibodies

### 5.1. Modeling Immunomodulatory Functions 

Antibodies can activate and engage innate immune cells through the Fc-FcγR interactions. The activated effector cells, majorly natural killer (NK) cells, can release perforin or granzyme B to lyse the target cell. This effect is also known as antibody-dependent cellular cytotoxicity (ADCC) [229,230]. Another Fc-mediated effector function is antibody-dependent cellular phagocytosis (ADCP), by which antibody-opsonized target cells can activate macrophages and induce phagocytosis, leading to target cell degradation through phagosome acidification. Antibodies can also engage the complement system to trigger complement-dependent cytotoxicity (CDC). The Fc region recruits the complement cascade via interacting with C1q, ultimately leading to the targeted cells’ apoptosis. These Fc-dependent effector mechanisms are crucial for many marketed antibodies, especially for anti-tumor antibodies [231]. Although broadly evidenced in vitro, these mechanisms have incompletely understood participation in efficacy in vivo, which vary across antibodies, target antigens, tumor types, and patient populations [229,232,233,234,235]. For example, Cartron et al. demonstrated that the increased FcγR affinity to human IgG1 was associated with enhanced responses to rituximab in follicular lymphoma patients [234]. Trivedi et al. showed that anti-EGFR antibodies could activate multiple cellular immune responses, involving NK cells, neutrophils, and dendritic cells. These multicellular immune responses are critical for anti-EGFR antibodies in head and neck cancer patients [235]. Although similar EGFR signaling suppression, panitumumab is less effective in activating these cellular immune responses than cetuximab [235]. However, the relative participation of Fc-mediated effector functions in the treatment effects is not clear.

Antibodies such as immune checkpoint blockades (ICBs) can deploy the host adaptive immune system via blocking the immune checkpoint inhibitors, such as CTLA-4 and PD-1/PD-L1, enabling T-cell activation and proliferation [236,237]. The treatment efficacy of ICBs is highly dependent on the tumor immune environments, and the tumors with high T cell infiltrations and high expressions of these checkpoint inhibitors tend to respond better to ICBs [238]. The locally rejuvenated or peripherally active T cells can both greatly contribute to the effect of ICBs. Recent studies have suggested that the anti-tumor immunity that newly recruited from the periphery may have greater contributions to the response than locally reinvigorated immunity [239,240,241]. For antibodies with systemic effects, namely the ability to recruit immune cells from the periphery lymphatic systems, antibody distribution at the primary target locations may not significantly influence the treatment effects. One recent study showed that metastases at multiple anatomical sites responded to a similar degree to pembrolizumab, even though these anatomical sites could have different antibody distribution degrees [95,242,243]. There is an increasing need to use the modeling approach to elucidate the roles of tissue disposition and immune functions in ICB treatment effects.

One major challenge to develop mechanism-based PD models for antibodies is the lack of techniques to longitudinally assessing the immunomodulatory functions in the living system, particularly in the evolving TME [244,245]. In vitro measurements of immunomodulatory functions are mainly for mechanistic exploration and are limited in predicting the in vivo immune dynamics due to the lack of physiological contexts [79,246]. Many biomarkers only provide a time-frozen snapshot of the immune status but fail to reveal the dynamically immune functions [247]. Longitudinally monitoring the immune signatures in tumor samples could yield insights into the response and resistance mechanisms to ICBs [248]. Liu et al. developed a dynamic matrix-based biomarker that integrates the interferon γ cytokine secretion as a biomarker to predict the immunomodulatory effects of anti-PD-1 antibodies [249]. Litchfield et al. performed a pan-tumor analysis to reveal the relative importance of tumor-cell-intrinsic and TME features underpinning ICB responses and resistance [250]. Despite these efforts, it is still challenging to accurately project the effector functions in vivo and the pharmacodynamic responses of immunotherapies.

### 5.2. Modeling the Resistance to Antibody Treatments

Despite the great success, antibody therapies are deeply challenged by treatment resistance. The resistance is primarily rooted in the disparity between antibody target selectivity and heterogeneous disease genotypes and phenotypes. The success of antibodies in a fraction of patients makes it compelling to interrogate antibody efficacy and resistance in specific physiological contexts in the hope of expanding effectiveness to the broader population.

There are innate resistance and acquired resistance to antibody therapies. Elucidating the molecular basis of resistance is critical for making actionable strategies to prevent or treat them. The expression of a certain set of genes enriched in patients who do not respond to antibody therapies can help us define the molecular features of resistance. The tumor-intrinsic and extrinsic factors associated with treatment resistance have been broadly reviewed before [251,252,253]. The acquired resistance to antibody therapy partly results from intra-tumoral heterogeneity and clonal evolution. Therapeutic antibodies pose high selective pressures to tumor cells eradicating the sensitive cell populations but enriching the clones with high resistance potentials with high efficiency [254,255,256]. As the antibodies deplete the sensitive cell population, the feature of TME shifts toward the outgrowth of the alternative populations that are less sensitive to the antibody treatment yet out-competed by the previous incumbent populations before antibody treatment. This dynamic shift in the cell populations may advance polyclonal resistance to therapeutic antibodies [257].

Modeling tumor resistance using evolutionary theories is helpful for predicting the resistance trajectories and exploring possibilities to overcome them [256,258,259]. Many modeling works characterized cancer genetic and clinical progression as a stochastic process [260], in which tumor cells proliferate, divide, and apoptosis based on probabilities [261,262,263,264,265]. Dynamic tumor evolution models were developed to predict the treatment responses of therapeutic antibodies [266,267,268,269,270,271,272,273,274]. For instance, Zhou et al. developed a parsimonious evolutionary modeling framework to analyze longitudinal development of bulk tumors prior to diagnosis and during and after therapy [269]. In this study, the evolutionary model was developed based on longitudinal development of liver metastatic lesions in metastatic colorectal cancer (mCRC) patients. The stochastic model recapitulated individual patient evolutionary dynamics, which could predict clinical outcomes in mCRC patients. Foo et al. evaluated the influences of PK parameters and dosing schedules on the evolution of T790M-mediated cancer resistance [273]. Those works demonstrated the potentials to estimate the probability of success for different treatment regimens by modeling approaches.

## 6. Conclusions

Therapeutic antibodies have achieved remarkable success in treating many diseases. However, therapeutic antibodies still face many outstanding issues associated with their PK and PD, including high variabilities, low tissue distributions, poorly-defined PK/PD characteristics for novel antibody formats, and high treatment resistance. Here we reviewed the state-of-art bioanalytical approaches for assessing antibody tissue distribution and target engagement in living animals. The unique aspects in modeling the PK/PD of emerging antibodies, such as recycling antibodies, bsAbs, and antibody-related gene therapies, were also highlighted. The increasing roles of PK/PD modeling in antibody discovery, preclinical development, and translational research were thoroughly discussed. Further directions for therapeutic antibodies were underlined, including elucidating the sources of high PK/PD variability, developing strategies for enhancing antibody delivery into solid tumors and the brain, designing new formats for improved target engagement and effector functions, and developing prognostic biomarkers for clinical resistance and response. Mechanism-based PK/PD models could be critical to address these challenges for therapeutic antibodies.
